# Lattice Expansion in Rb-Doped Hybrid Organic–Inorganic Perovskite Crystals Resulting in Smaller Band-Gap and Higher Light-Yield Scintillators

**DOI:** 10.1021/acs.inorgchem.3c00270

**Published:** 2023-05-26

**Authors:** Francesco Maddalena, Muhammad Haris Mahyuddin, Dominik Kowal, Marcin E. Witkowski, Michal Makowski, Md Abdul Kuddus Sheikh, Somnath Mahato, Roman Jȩdrzejewski, Winicjusz Drozdowski, Christophe Dujardin, Cuong Dang, Muhammad Danang Birowosuto

**Affiliations:** † School of Electrical and Electronic Engineering, Nanyang Technological University, Singapore 639798, Singapore; ‡ CINTRA UMI CNRS/NTU/THALES, 3288 Research Techno Plaza, 50 Nanyang Drive, Border X Block, Level 6, Singapore 637553, Singapore; § Research Group of Advanced Functional Materials, Faculty of Industrial Technology, Institut Teknologi Bandung, Bandung 40132, Indonesia; ∥ Lukasiewicz Research Network-PORT Polish Center for Technology Development, Stablowicka 147, Wroclaw 54-066, Poland; ⊥ Institute of Physics, Faculty of Physics, Astronomy, and Informatics, Nicolaus Copernicus University in Torun, ul. Grudziadzka 5, Torun 87-100, Poland; ∇ Universitè de Lyon, Universitè Claude Bernard, Lyon 1, CNRS, Institut Lumière Matière UMR5306, Villeurbanne F-69622, France

## Abstract

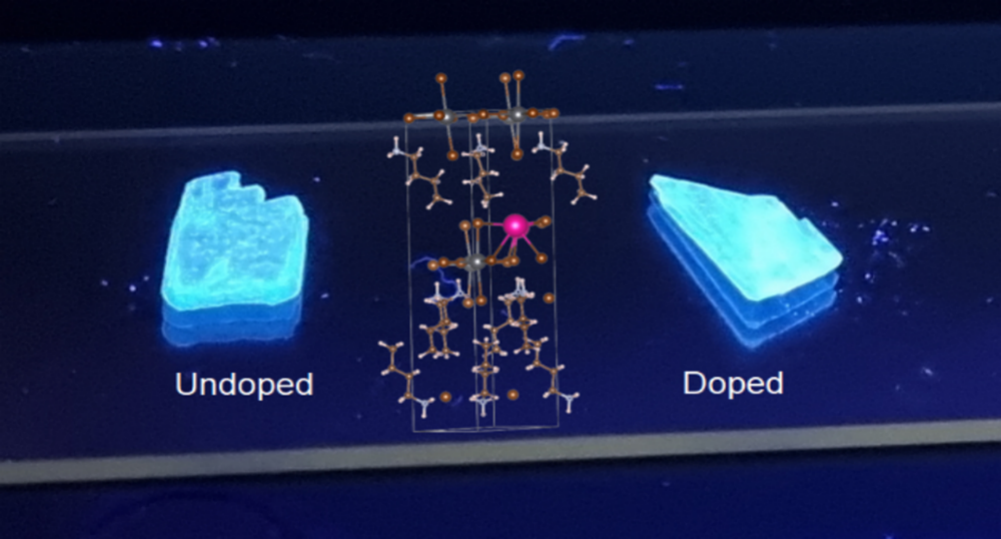

Two-dimensional hybrid-organic–inorganic perovskite (2D-HOIP) lead bromide perovskite crystals have demonstrated great potential as scintillators with high light yields and fast decay times while also being low cost with solution-processable materials for wide energy radiation detection. Ion doping has been also shown to be a very promising avenue for improvements of the scintillation properties of 2D-HOIP crystals. In this paper, we discuss the effect of rubidium (Rb) doping on two previously reported 2D-HOIP single crystals, BA_2_PbBr_4_ and PEA_2_PbBr_4_. We observe that doping the perovskite crystals with Rb ions leads to an expansion of the crystal lattices of the materials, which also leads to narrowing of band gaps down to 84% of the pure compounds. Rb doping of BA_2_PbBr_4_ and PEA_2_PbBr_4_ shows a broadening in the photoluminescence and scintillation emissions of both perovskite crystals. Rb doping also leads to faster γ-ray scintillation decay times, as fast as 4.4 ns, with average decay time decreases of 15% and 8% for Rb-doped BA_2_PbBr_4_ and PEA_2_PbBr_4_, respectively, compared to those of undoped crystals. The inclusion of Rb ions also leads to a slightly longer afterglow, with residual scintillation still being below 1% after 5 s at 10 K, for both undoped and Rb-doped perovskite crystals. The light yield of both perovskites is significantly increased by Rb doping with improvements of 58% and 25% for BA_2_PbBr_4_ and PEA_2_PbBr_4_, respectively. This work shows that Rb doping leads to a significant enhancement of the 2D-HOIP crystal performance, which is of particular significance for high light yield and fast timing applications, such as photon counting or positron emission tomography.

## Abstract 2

Two-dimensional hybrid-organic−inorganic perovskite lead bromide perovskite crystals have demonstrated great potential as scintillators with high light yields and fast decay times while also being low cost with solution-processable materials for wide energy radiation detection. This work shows that Rb doping leads to a significant enhancement of the 2D-HOIP crystal performance, which is of particular significance for high light yield and fast timing applications, such as photon counting or positron emission tomography.

## Introduction

Two-dimensional hybrid-organic–inorganic perovskite (2D-HOIP) crystals have recently been shown to be excellent candidates for radiation and particle detection, exhibiting high light yields, negligible afterglow, and short scintillation decay times,^1−7^ making these materials very attractive both for X-ray imaging and for fast timing applications.^8^ In addition, 2D-HOIPs generally possess high stability even under environmental conditions, in particular, oxygen and moisture, compared to other hybrid perovskite materials and many other scintillating materials. Furthermore, 2D-HOIPs can be synthesized and processed from solution at low temperatures (*T* < 200 °C) and without the need for high-vacuum technology.^2,5,9^ This makes 2D-HOIPs synthesis and deposition less energy intensive, hence leading to lower production cost and potentially allowing for large area deposition techniques such as roll-to-roll printing. The 2D-HOIPs BA_2_PbBr_4_ and PEA_2_PbBr_4_, in particular, have been shown to be particularly environmentally robust and to have high light yields up to record values of 40 000 and 11 000 photons/MeV, respectively,^5,10^ and energy resolution below 10% at 662 keV. Recently, we have demonstrated the use of PEA_2_PbBr_4_ for imaging and particle detection applications,^5,10^ and we have shown that lithium (Li) cation doping leads to a significant improvement of the scintillation of BA_2_PbBr_4_ and PEA_2_PbBr_4_, specifically increased light yield and better energy resolution.^10,11^

Here, we explore the effects of rubidium (Rb) doping on the BA_2_PbBr_4_ and PEA_2_PbBr_4_ 2D-HOIP crystals. The choice of Rb-ion doping was motivated by the fact that several works have shown that Rb-ion doping of perovskites leads to an improvement of their optoelectronic properties.^12−15^ The larger effective ionic radius^16−19^ of (6-coordinate) Rb^+^ (152 pm) in comparison to that of (6-coordinate) Li^+^ (76 pm) may have stronger effects in the optical and scintillation properties of both 2D-HOIP crystals.^11^ For this purpose, we synthesized undoped and Rb-doped BA_2_PbBr_4_ and PEA_2_PbBr_4_ single crystals, and we characterized the structure using X-ray diffraction (XRD) and confirmed the inclusion of Rb using X-ray photoemission spectroscopy (XPS). Based on the XRD results, we conducted density functional theory (DFT) calculations of the band structure and density of states (DOS) of the materials. We carried out optical characterization of the undoped and Rb-doped HOIP crystals, both absorption and photoluminescence (PL) measurements, in order to observe the effects of the Rb doping on the 2D-HOIP crystals. We also carried out temperature-dependent radioluminescence (RL) measurements to explore the effects of the inclusion of Rb on the scintillation properties of the perovskite, including the effect on the afterglow. To study the trap levels in the undoped and Rb-doped 2D-HOIP crystals, we conducted thermoluminescence (TL) measurements. Finally, we measured γ-ray pulse height spectra (PHS) using ^241^Am (*E*_γ_ = 59.5 keV) to determine the effect of Rb doping on the light yield of the 2D-HOIP crystals. From our characterizations, we observe that Rb doping of the BA_2_PbBr_4_ and PEA_2_PbBr_4_ crystals leads to an expansion of the lattice cells. This effect is more prominent in BA_2_PbBr_4_. The lattice expansion leads to smaller band gaps for the 2D-HOIP crystals with a decrease of 0.57 and 0.12 eV for the Rb-doped BA_2_PbBr_4_ and PEA_2_PbBr_4_, respectively, compared to the undoped counterparts. In addition, doping also leads to a broadening of the PL and RL spectra, especially for PEA_2_PbBr_4_. Faster PL and RL decay times in addition to additional TL peaks indicate that the presence of the Rb ion in the structure leads to the formation of additional trap states. However, Rb doping also leads to significant improvements of the light yields with increases of 58% and 25% for BA_2_PbBr_4_ and PEA_2_PbBr_4_, respectively, while the scintillation decay times are faster, 15% and 8% for BA_2_PbBr_4_ and PEA_2_PbBr_4_, respectively. Although the 58% increase is still smaller than the 78% increase of Li-doped PEA_2_PbBr_4_,^11^ the current study has better insight on how the ion doping may cause the lattice expansion and the narrowing band gap resulting in the light yield increase and the scintillation decay time decrease. The augmented light yield and faster scintillation decay indicate that Rb doping of 2D-HOIP crystals is very promising as a tool to improve the performance of 2D-HOIPs, in particular, for application in fast timing imaging applications and radiation detection.

## Experimental Section

### Perovskite Crystal Synthesis

BA_2_PbBr_4_ and PEA_2_PbBr_4_ crystals were prepared using a modified version of the previously used solution method.^10,11^ Dimethyl sulfoxide (DMSO, anhydrous), butylammonium bromide ((BA)Br, ≥98%)), phenethylammonium bromide ((PEA)Br, ≥98%), lead bromide (PbBr_2_, ≥98%), and Rb bromide (RbBr, ≥98%) were purchased from Sigma-Aldrich and used without further purification. A 3 M precursor solution was prepared by dissolving (BA)Br or (PEA)Br and PbBr_2_ in stoichiometric amounts in DMSO under stirring at 100 °C for 2 h. For the Rb-doped perovskite, RbBr was also added to the solution, Rb:Pb = 5:100. Crystals were obtained by letting DMSO slowly evaporate from the precursor solutions in ambient environment for a period of about 30 days. The crystal precipitate was then washed with hexane and dried under vacuum for future characterization.^10,11^

### High-Resolution X-ray Diffractometry

XRD experiments were performed on the best 2D-HOIP crystals. To collect the diffraction patterns, we used a high-resolution X-ray diffractometer (Empyrean, PANalytical) equipped with a hybrid monochromator on the incident beam path and a Pixcel3D detector on the diffracted beam path. The measurements were performed using Cu Kα1 (1.540591 Å) emissions. The angular resolution of 2θ was 0.0002°. The diffraction patterns were collected after crystals alignment in the range from 5° to 65° 2θ.

### Composition Characterization

XPS measurements were performed using a Kratos AXIS Supra X-ray Photoelectron Spectrometer equipped with monochromatic Al Kα radiation (*E*_*ex*_ = 1486.6 eV). The analysis area was approximately 700 × 300 μm. Charge correction was done by referencing the adventitious C–C peak to 284.8 eV.

### DFT Calculations

The calculations were performed under the Kohn–Sham formulation^20,21^ as implemented in the Vienna Ab-initio Simulation Package (VASP).^22^ The projector augmented wave (PAW) method^23,24^ was used to describe the interaction between the ion cores and the electrons. The electron exchange-correlation was treated by the generalized gradient approximation (GGA) based on the Perdew–Burke–Ernzerhof (PBE) functional. The rotationally invariant GGA+U approach introduced by Dudarev et al.^25^ was used with the effective Hubbard parameter *U*_eff_ being 4.0 eV for Pb p orbitals. The plane wave basis sets with a cutoff energy of 500 eV were used for all calculations. The Brillouin zones with *k*-point grids of 5 × 5 × 1 and 3 × 3 × 3 for respective BA_2_PbBr_4_ and PEA_2_PbBr_4_ structures were used according to the Monkhorst–Pack scheme.^26^ The zero-damping D3 method^27^ was adopted to account for the dispersion correction. During calculations, all atoms were allowed to fully relax. The conjugate gradient method was employed for cell optimization, and the calculations were considered to converge when the maximum forces on each atom were less than 0.01 eV/Å.

### PL and Absorption Measurements

PL measurements were performed using a custom-built micro-PL setup at 300 K and ambient atmosphere. Pulsed lasers (PicoQuant) with an ultraviolet (UV) excitation of 355 nm wavelength with a pulse width of 15 ps and a 10 MHz repetition rate were focused on samples by a microscope objective (Olympus, 40× objective, NA = 0.65, and focused laser beam diameter ≈ 1 μm). The PL spectra were collected using a thermoelectric-cooled Avaspec HERO spectrometer. The time-resolved PL (TRPL) decay curves were collected using a 375 nm laser with a 200 kHz repetition rate and photomultiplier (PMT) tube (Hamamatsu H7422 series). Finally, absorption spectra were measured with a homemade setup with a commercial Avaspec spectrometer in transmission mode. Since the samples were put inside the quartz tubes, the spectra were corrected for the absorption of the tubes.

### RL and TL Measurements

For all measurements, we used one integrated setup. It consists of an Inel XRG3500 X-ray generator Cu-anode tube, 45 kV/10 mA, an Acton Research Corp. SpectraPro-500i monochromator, a Hamamatsu R928 photomultiplier tube (PMT), and an APD Cryogenic Inc. closed-cycle helium cooler. First, we recorded low-temperature afterglow at 10 K by exposing the crystals to X-rays for 10 min. Then, we measured TL glow curves at temperatures between 10 and 350 K with a heating rate of about 0.14 K/s. Afterward, we measured RL spectra at different temperatures between 350 and 10 K starting from the highest to the lowest temperatures in order to avoid possible contributions from thermal release of charge carriers to the emission yield.

### PHS Measurements

^241^Am (59.5 keV) and ^137^Cs (661.7 keV) radioisotope γ-ray sources and a Hamamatsu PMT R878 were used to detect the converted photons. The PMT was operated at a voltage of 1250 V. The corresponding output signal from the PMT is integrated with a charge-sensitive preamplifier (Canberra 2005). The output then feeds a spectroscopic amplifier with a shaping time of 2 μs (Canberra 2022), which finally is processed by a TUKAN-8K-USB multichannel analyzer.

## Results and Discussion

**Figure 1:**
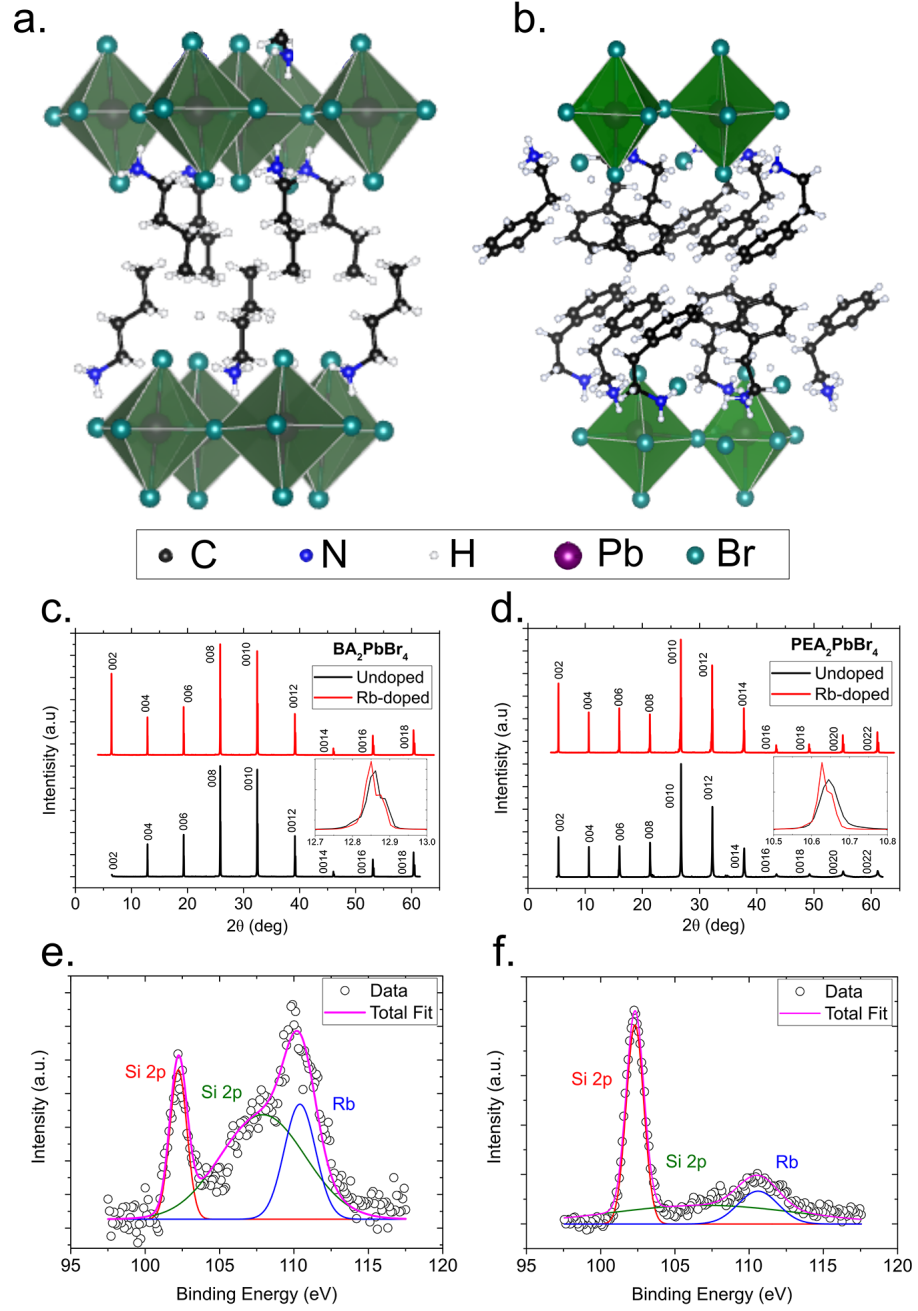
Crystal structure, XRD, and XPS of undoped and Rb-doped BA_2_PbBr_4_ and PEA_2_PbBr_4_. Crystallographic structure of (a) BA_2_PbBr_4_ and (b) PEA_2_PbBr_4_. X-ray difractograms of undoped and Rb-doped (c) BA_2_PbBr_4_ and (d) PEA_2_PbBr_4_. The inset shows the shift in the *hkl* = [0 0 4] peak between the undoped and the Rb-doped perovskite. XPS Rb signal for Rb-doped (e) BA_2_PbBr_4_ and f() PEA_2_PbBr_4_ and the corresponding fit.

The structures of the 2D-HOIP crystals are shown in Figure 1a and 1b for BA_2_PbBr_4_ and PEA_2_PbBr_4_, respectively. The crystals feature a Ruddlesden–Popper layered structure,^11^ where single layers of PbBr_6_^4–^ octahedra are separated by a double layer of organic monovalent cations. From single-crystal XRD spectra (Figure 1c and 1d), we analyzed the perovskite structures for both undoped and Rb-doped materials. Using Rietveld refinement on the XRD data (Supplementary Figure S1), we analyzed the effects of the incorporation of Rb in the perovskite structure. The results of the analysis have been tabulated in Supplementary Table S1. For pure BA_2_PbBr_4_, our volume is only about ±0.5% larger than the average volumes reported in refs (28−34), while the previous XRD results are mostly measured as microcrystals or powder forms. Meanwhile, for PEA_2_PbBr_4_, the lattice parameters are very similar to those reported in refs (31) and (35). The diffractograms for Rb-doped materials also reveal that when Rb is incorporated into the perovkites, by substitution of some of the organic cations (Supplementary Figure S1), expansion of the lattice occurs compared to the undoped 2D-HOIP crystals. The lattice volume of BA_2_PbBr_4_ increases from 1899 Å^3^ for the undoped crystal to 1912 Å^3^ for the Rb-doped perovskite one, corresponding to a 0.68% increase. Here, we note that the BA_2_PbBr_4_ crystals are the same batches, while we are sure that there are no fluctuations from the same sample types. Even the value of 1912 Å^3^ in Rb-doped BA_2_PbBr_4_ is still larger, 0.42%, than that reported in ref (34). Similarly, that of PEA_2_PbBr_4_ increases from 2247 Å^3^ for the undoped crystal to 2255 Å^3^ for the Rb-doped one, corresponding to a 0.36% increase. This expansion results in a slight shift of the peaks in the diffraction pattern, as seen in the insets of Figure 1c and 1d. In addition, for BA_2_PbBr_4_, the incorporation of Rb ions leads to a change of the cell structure, from orthorhombic to triclinic, at room temperature. The structure of PEA_2_PbBr_4_ is triclinic for both undoped and Rb-doped variants. As a reference with another Rb perovskite crystal, RbPb_2_Br_5_, its cell structure is body-centered cubic and its volume is smaller in comparison to the above-mentioned Rb-doped 2D-HOIP crystals as shown in Supplementary Figure S2 and Supplementary Table S1, respectively. To confirm the presence of Rb in the doped 2D-HOIP crystals, we also carried out XPS measurements. Figure 1e and 1f shows the Rb peaks. The Br, C, N, and Pb peaks are presented in Supplementary Figure S3. We clearly see the signal of the Rb 3d orbital at a binding energy of 110 eV, consistent with Rb–Br interaction.^36^ We also observe further binding energy peaks at 102 and 108 eV, which arise from the Si 2p orbital from traces of silicon^36^ present in the substrate used in the measurement. From the ratio of the integrals between the Rb 3d and the Pb 4f peaks, we obtain that the concentration of Rb is slightly lower than 5% for both crystals which is consistent with the results from inductively coupled plasma mass spectrometry, see Supplementary Table S1. Finally, Raman spectra in Supplementary Figure S4 show the influence of the doping on the shifts in the vibrational bands. Such shifts may indicate that the doping ion may replace one of the ion sites in the organic ligands.^37^

**Figure 2:**
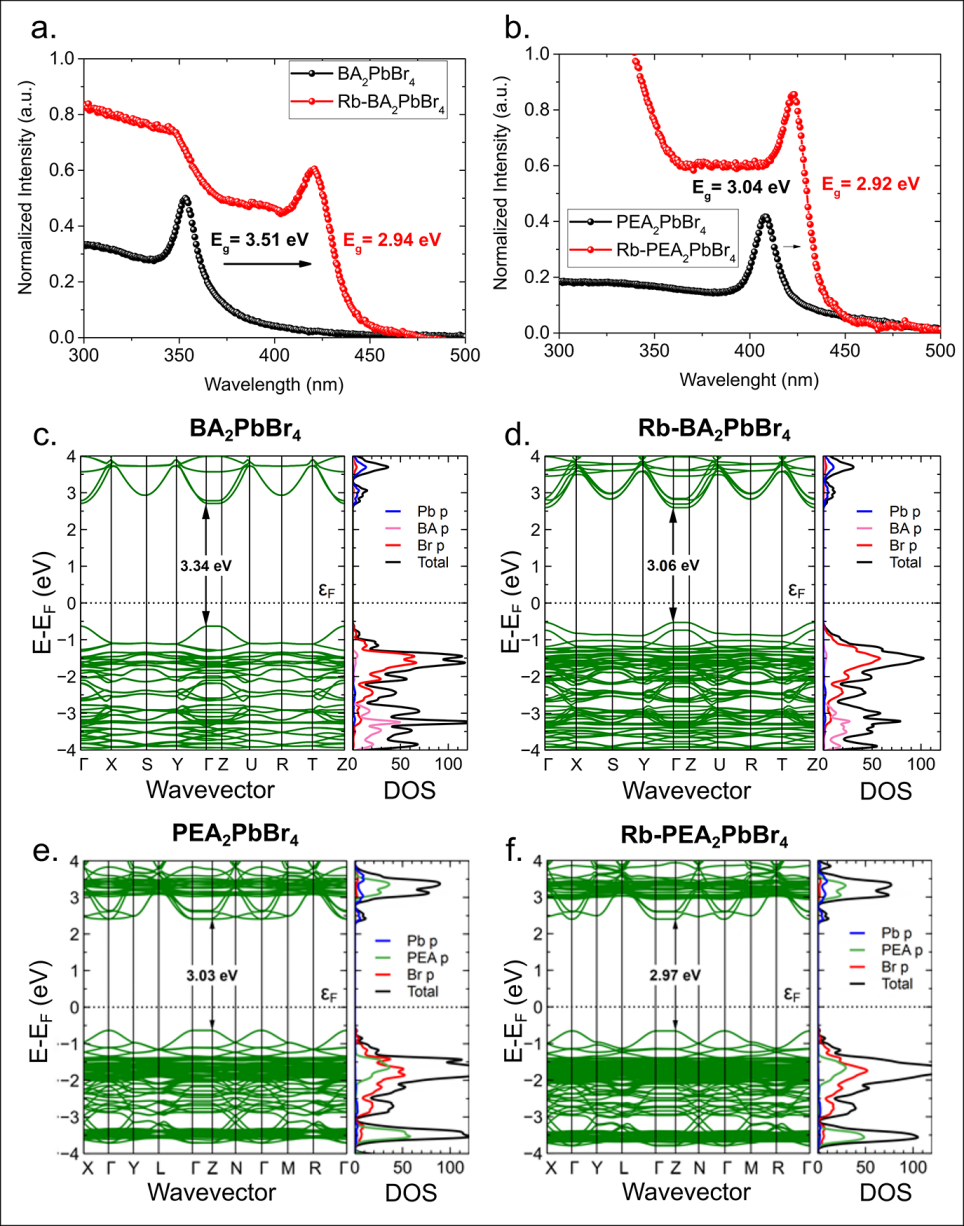
Absorption spectra, band structure, and density of states. Absorption spectra of undoped and Rb-doped (a) BA_2_PbBr_4_ and (b) PEA_2_PbBr_4_. Band structure and density of states of (c) undoped and (d) Rb-doped BA_2_PbBr_4_ and (e) undoped and (f) Rb-doped PEA_2_PbBr_4_. The numbers indicated in the band structure define band-gap values similar to those obtained from the absorption spectra.

Figure 2a and 2b shows the absorption spectra for the undoped and Rb-doped BA_2_PbBr_4_ and PEA_2_PbBr_4_ 2D-HOIP crystals, respectively. We observe a shift in the spectra and fit the data using the Elliot formalism^38^ (Supplementary Figure S5), The spectra show narrowing of the band gaps for both Rb-doped BA_2_PbBr_4_ and PEA_2_PbBr_4_ in comparison to those of the undoped variants.^39^ The band gap of BA_2_PbBr_4_ decreases from 3.51 to 2.94 eV while that of PEA_2_PbBr_4_ decreases from 3.04 to 2.92 eV for the undoped and Rb-doped samples, respectively. The effect of the Rb ions on the absorption is stronger in BA_2_PbBr_4_, despite similar concentrations for both perovskite types. This is likely due to the closer spacing between the lead bromide octahedral layers.^5^ The closer spacing might result in a stronger influence of Rb ions on the optoelectronic properties of the perovskite, as seen in the Raman spectra (Supplementary Figure S4). The smaller values in Rb-doped 2D-HOIP crystals are consistent if we consider a typical band gap of another Rb perovskite crystal, RbPb_2_Br_5_, of 2.01 eV (Supplementary Figure S2). The narrowing of the band gap is also consistent with the expansion of the lattice cell of the 2D-HOIP crystals due to the inclusion of Rb ions, as previously observed in other doped halide perovskites,^40,41^ as the energy gap is inversely proportional to the dielectric constant which in turn is inversely proportional to the interatomic distance. This leads to the valence and conduction bands shifting closer to the Fermi level, as corroborated by our density functional theory (DFT) calculations using the cell structure parameters determined from our XRD measurements from Supplementary Table S1. We show the results of the DFT calculations, both the band structure and the DOS, in Figure 2c–f. The shifts of the band gaps from the calculations for Rb-doped BA_2_PbBr_4_ and PEA_2_PbBr_4_ are 0.28 and 0.06 eV, respectively, and they still agree with those from the experiments of 0.57 and 0.12, respectively. We note that this lattice expansion and reduction of the band gap is further evidence against the possibility that the Rb ions replace Pb ions in the structure, since they would lead to significant distortion of the PbBr_6_ octahedra layers and corresponding widening of the band gap.^12,18,42^ Furthermore, although Rb ions can be both substituents in the A site (the organic cation) and an interstitial defect,^43^ the red shift in the absorption spectra, the results from our XRD analysis, and the overall narrowing of the band gap strongly suggests that Rb ions are not simply placed in interstitial positions but replace the organic cations, leading to the observed lattice expansion. Raman spectroscopy measuremenrts (Supplementary Figure SX) also show the Raman shift with vibrational modes consistent with Rb ions substituting the organic cations.^44^ Since Rb doping leads to a narrower band gap, we expect an increase in the light yield as well. The light yield (*LY*) of a material is inversely proportional to the band gap, *LY* ≈ *S*·*Q*/*E*_g_, where *S* is the charge transport parameter, *Q* is the quantum efficienty, and *E*_g_ is the band gap.^1,2^ However, we also note that in practice the light yield increase may not be linearly proportional to the band gap decrease, since Rb doping may also affect the quantum yield and charge transport in the material as well. Finally, the DFT calculations reveal that doping does not otherwise strongly alter the band structure and the DOS of the 2D-HOIP crystals, and all structures show a direct band-gap transition.

**Figure 3:**
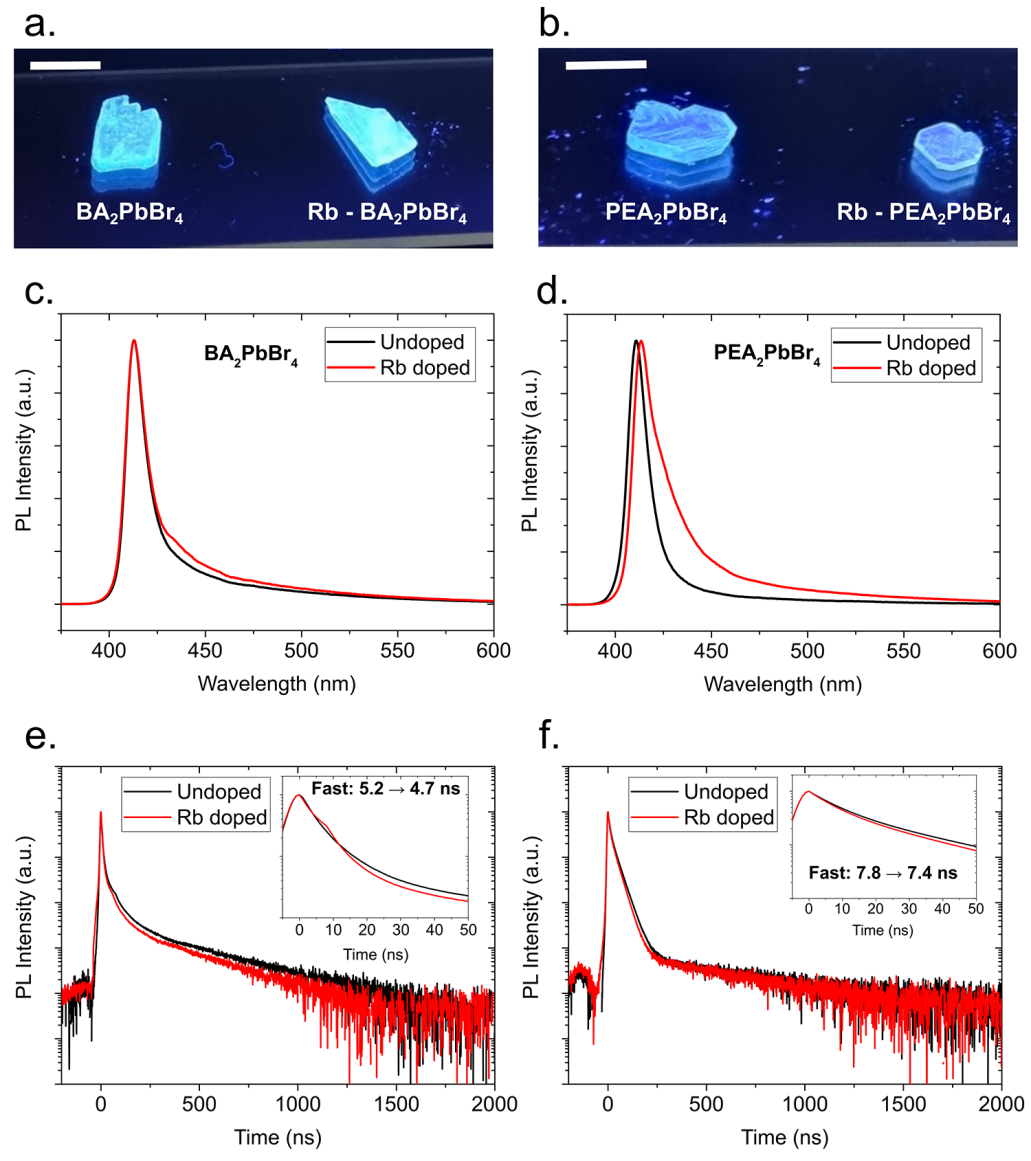
Emission images under UV light (λ_ex_ = 371 nm) for undoped and Rb-doped (a) BA_2_PbBr_4_ and (b) PEA_2_PbBr_4_ crystals. Photoluminescence (PL) spectra (λ_*ex*_= 355 nm) for undoped and Rb-doped (a) BA_2_PbBr_4_ and (b) PEA_2_PbBr_4_. Time-resolved PL decay (λ_ex_ = 355 nm) for undoped and Rb-doped (c) BA_2_PbBr_4_ and (d) PEA_2_PbBr_4_. The insets show zoomed in regions in of the PL decay curves between 0 and 50 ns and the values of the fast PL decay time components.

Figure 3a and 3b shows the emission images of undoped and Rb-doped BA_2_PbBr_4_ and PEA_2_PbBr_4_ single crystals under UV illumination (λ_ex_ = 371 nm), respectively. All crystals show a bright emission in the blue region, and by eye, the emission of the Rb-doped BA_2_PbBr_4_ crystal is brighter than the undoped BA_2_PbBr_4_ one, while both undoped and Rb-doped PEA_2_PbBr_4_ seem to have the same brightness. The PL emission spectra are reported in Figure 3c for undoped and Rb-doped BA_2_PbBr_4_. The peak emission wavelength (412 nm) does not change when Rb ions are introduced in the perovskite structure; however, there is a minor broadening in the emission, as BA_2_PbBr_4_ shows a full-width half-maximum (fwhm) value of 12.7 nm (91.9 meV) for the undoped variant and 13.3 nm (96.2 meV) for the Rb-doped variant. The Rb-doped BA_2_PbBr_4_ also shows in increased emission at wavelengths > 425 nm. In contrast, PEA_2_PbBr_4_ shows a slight shift in peak emission from 411 to 413 nm for the undoped and Rb-doped 2D-HOIP crystals, respectively. The addition of Rb ions to the PEA_2_PbBr_4_ structure also leads to a significant broadening of the emission. PEA_2_PbBr_4_ shows a fwhm value of 12.1 nm (88.9 meV) for the undoped variant and 18.5 nm (132 meV) for the Rb-doped variant. The broadening of the emissions, in particular, toward longer wavelengths, is consistent with the lowered band gap in the neighborhood of the Rb ion dopants, and inhomogeneous broadening^45−47^ is introduced by the distortion and defects of the crystal lattice due to lattice expansion in the neighborhood of Rb ions compared to the Rb-free regions. The larger broadening observed in the Rb-doped PEA_2_PbBr_4_, compared to the minor broadening in BA_2_PbBr_4_, indicates that overall the structure of PEA_2_PbBr_4_ is more prone to defects despite the volume change in the lattice cell being less pronounced in PEA_2_PbBr_4_. The introduction of Rb ions into the perovskite structure also leads to faster PL decay times. The time-resolved PL curves are shown in Figure 3e and 3f for undoped and Rb-doped BA_2_PbBr_4_ and PEA_2_PbBr_4_, respectively, and the three exponential fitting parameters are shown in Supplementary Table S2. The fast decay time component of BA_2_PbBr_4_ goes from 5.2 to 4.7 ns for the undoped and Rb-doped 2D-HOIP crystals, respectively. Similarly, the fast decay time component of PEA_2_PbBr_4_ goes from 7.8 to 7.4 ns for the undoped and Rb-doped 2D-HOIP crystals, respectively. The addition to Rb ions to the structure also causes the contribution of the fast component to become slightly more prominent and the contribution of the slowest component to the decay to decrease, especially for BA_2_PbBr_4_. In contrast with Li-doped 2D-HOIP crystals,^11^ this effect only appears on PEA_2_PbBr_4_ but not with BA_2_PbBr_4_. Because of Rb doping, the average decay time of BA_2_PbBr_4_ has been improved from 74.6 to 51.2 ns while that of PEA_2_PbBr_4_ has been improved from 56.2 to 41.7 ns. The percentages of improvements are 45% and 35% for BA_2_PbBr_4_ and PEA_2_PbBr_4_, respectively.

**Figure 4:**
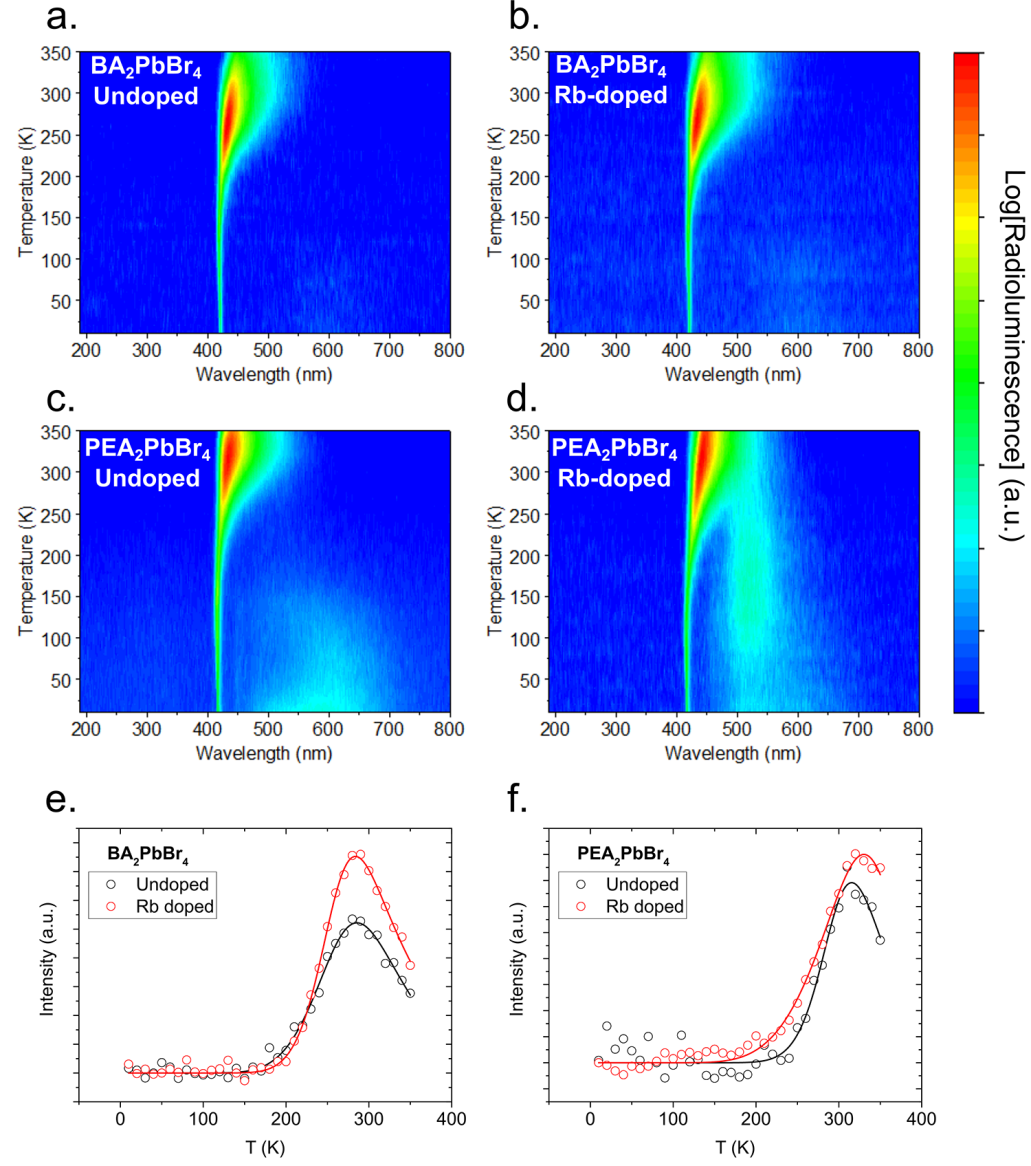
Radioluminescence (RL) and negative thermal quenching (NTQ). Temperature-dependent RL for (a) undoped and (b) Rb-doped BA_2_PbBr_4_ and (c) undoped and (d) Rb-doped PEA_2_PbBr_4_. Integrated RL for undoped and Rb-doped (e) BA_2_PbBr_4_ and (f) PEA_2_PbBr_4_. The solid lines represent the negative thermal quenching fit.

Figure 4a and 4b shows the temperature-dependent RL emission for undoped and Rb-doped BA_2_PbBr_4_, respectively, while Figure 4c and 4d shows the emission for undoped and Rb-doped PEA_2_PbBr_4_, respectively. The RL spectra of the undoped 2D-HOIP crystals are similar to what we previously reported,^5,10,11^ showing a very narrow RL signal at low temperatures. At 50 K, the RL emission spectra peak at 422 and 418 nm for BA_2_PbBr_4_ and PEA_2_PbBr_4_, respectively, and both show a fwhm ≈ 5 nm. Furthermore, at low temperatures, both 2D-HOIP crystals exhibit weaker but broader emissions centered at 603 and 590 nm for BA_2_PbBr_4_ and PEA_2_PbBr_4_, respectively. This feature is more prominent in PEA_2_PbBr_4_. This main emission broadens significantly and red shifts as the temperature increases. At 290 K, the peak emission shifts to 438 and 430 nm for BA_2_PbBr_4_ and PEA_2_PbBr_4_, respectively, with fwhm values of 30 and 25 nm. At 290 K, the RL emission is also red shifted compared to the PL emission as shown in Supplementary Figure S6, which is generally expected in scintillators.^2^

Rb doping of the 2D-HOIP crystals shows very similar emission characteristics to the undoped variants, as observed by comparing Figure 4a and 4b for BA_2_PbBr_4_ and Figure 4c and 4d for PEA_2_PbBr_4_. At low temperatures, the main narrow emission remains unaltered when Rb ions are introduced in the perovskite structure. However, the broad emission at longer wavelengths is altered for PEA_2_PbBr_4_.^48^ While Rb-doped BA_2_PbBr_4_ still features the emission centered around 603 nm, the change is negligible. PEA_2_PbBr_4_, on the other hand, shows a significant change. While at very low temperatures (10–50 K) we can still observe the broad emission centered around 590 nm, we can also see a further peak emerging, centered at 515 nm, which increases with temperature, peaking at 170 K and then decreasing again as the temperature rises. At higher temperatures, as seen in Supplementary Figure S6, the Rb-doped BA_2_PbBr_4_ does not significantly change compared to the undoped variant, except for a slight broadening. Alternatively, and consistent with the PL emission, the Rb-doped PEA_2_PbBr_4_ shows both a shift in the peak emission from 430 to 438 nm at 290 K and a significant broadening of the emission with the fwhm going from 25 to 41 nm.

The temperature-dependent RL intensity divided by the extrapolated intensity at 0 K, *I*(*T* → 0), is shown in Figure 4e and 4f for BA_2_PbBr_4_ and PEA_2_PbBr_4_, respectively. As previously observed,^10,11^ both 2D-HOIP crystals feature negative thermal quenching (NTQ), i.e., the increase of luminescence as the temperature increases.^49,50^ From the measurement we observe that there is only a small change in the temperature profile of the integrated RL between undoped and Rb-doped BA_2_PbBr_4_. Both feature a constant RL emission from 10 to 170 K, and then, the emission rises, peaks at 280 K, and then declines again as the temperature rises again. Undoped and Rb-doped PEA_2_PbBr_4_ also have a very close integrated RL emission profile in temperature. Both show a constant RL emission up from 10 to 200 K and then an increase, peaking at 310 and 330 K for the undoped and Rb-doped perovskite, respectively. This kind of behavior resembles the temperature-dependent behavior of the luminescence of II–VI or III–V semiconductor systems such as n-type ZnS or n-type GaAs.^51−53^ However, while in an inorganic semiconductor usually the peak emission occurs between 100 and 200 K, 2D-HOIP crystals, such as BA_2_PbBr_4_ and PEA_2_PbBr_4_, have their peak emission around room temperature. The parameters used for the fit are shown in Supplementary Table S3. While Rb doping seems to have only a small effect on the temperature-dependent RL emission, the inclusion of Rb ions leads to an increase in both the typical and negative thermal quenching coefficients and the respective activation energies for BA_2_PbBr_4_, while the exact opposite trend occurs for PEA_2_PbBr_4_, where both the coefficients and the energies decrease. Since the inclusion of Rb ions in the perovskite has the same effect for both the regular and negative thermal quenching and the overall result on the overall integrated RL emission, the main change we observe is that that the Rb-doped perovskites have a higher peak emission, *I*_max_/*I*(*T* → 0), compared to the undoped perovskites. This is similar to the previously observed changes induced by lithium doping.^10,11^

**Figure 5:**
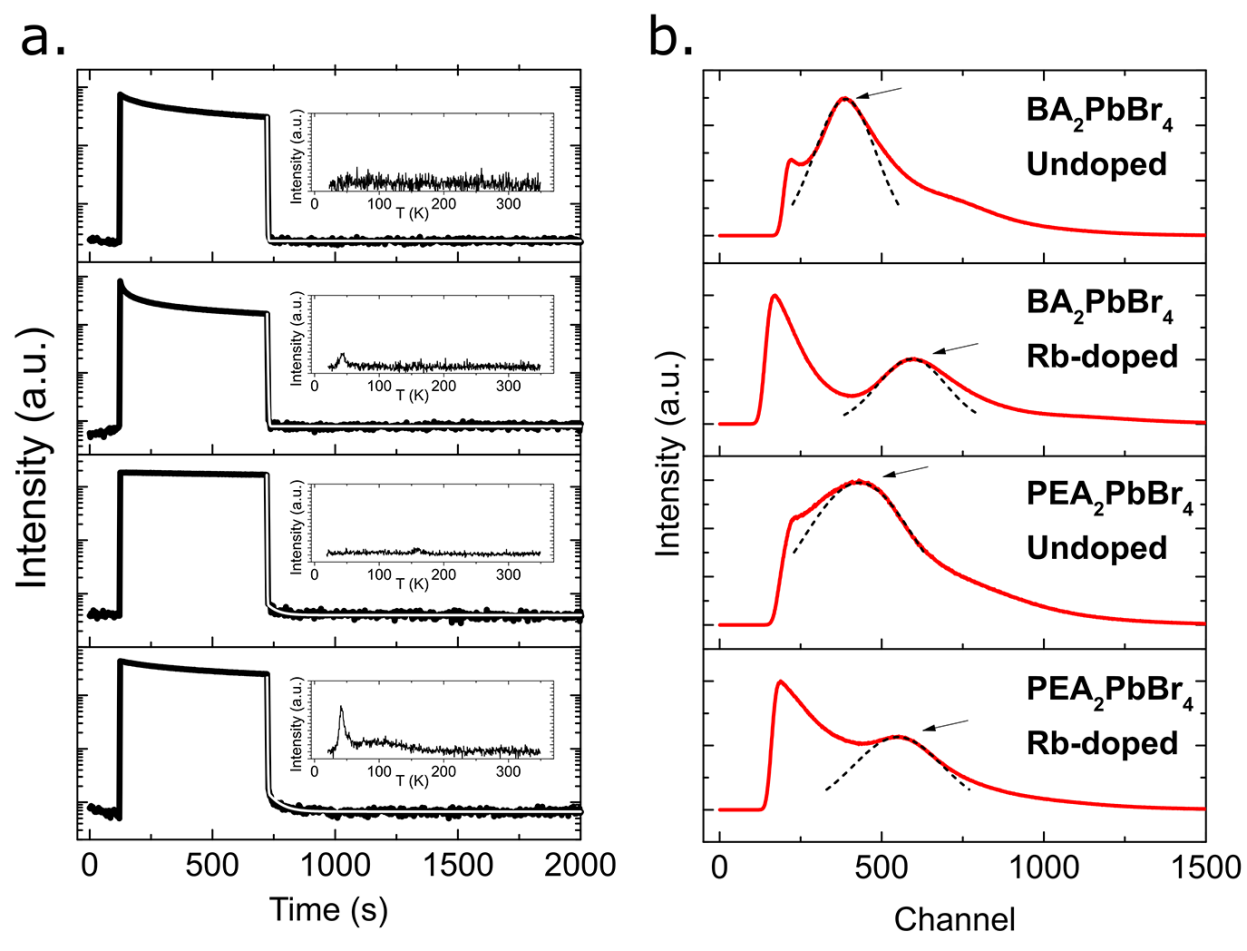
Afterglow and pulse height spectra (PHS) (from top to bottom) of undoped and Rb-doped BA_2_PbBr_4_ and undoped and Rb-doped PEA_2_PbBr_4_. (a) Afterglow measurement at *T* = 10 K for undoped and Rb-doped BA_2_PbBr_4_ and PEA_2_PbBr_4_, where the RL is held in the saturated regime for 10 min of X-ray exposure before the X-ray source is cut off. The white lines show the exponential decay fits. The insets in the figure show the thermoluminescence glow. (b) PHS with 59.5 keV (^241^Am) γ-ray sources for undoped and Rb-doped BA_2_PbBr_4_ and PEA_2_PbBr_4_. The arrows indicate the positions of the photopeaks, and the positions of the undoped peaks were normalized to each other to show the differences from the doped ones. The dashed line shows the Gaussian fit of the photopeaks.

To further investigate the effects of the inclusion of Rb ions into the 2D-HOIP structure, we conducted afterglow and TL measurements, as featured in Figure 5a. To measure the afterglow, i.e., the residual RL of the scintillator after exposure to X-rays, we exposed our 2D-HOIP crystals to the X-ray source, in the saturated regime of the RL, for 10 min at 10 K, which is the plateau observed in Figure 5a. The parameters of the exponential decay of the afterglow are shown in Supplementary Table S4. The effect of Rb doping of the 2D-HOIP crystals leads to an increase in the afterglow decay time for both BA_2_PbBr_4_ and PEA_2_PbBr_4_. BA_2_PbBr_4_ features a single-exponential decay of the afterglow with a decay time of 5.6 s. Rb-doped BA_2_PbBr_4_, on the other hand, features a double-exponential decay with decay times of 10.3 and 82.2 s and an average decay time of 22.3 s. Similarly, PEA_2_PbBr_4_ also features a monoexponential decay of the afterglow with a decay time of 0.9 s. Rb doping of PEA_2_PbBr_4_ introduces a double-exponential decay as well with decay times of 2.4 and 52.7 s and an average decay time of 5.1 s. Hence, we observe that the introduction of rubidium leads to both slower decay times and a more complex decay in general. The increase and the additional exponential component is probably caused by the introduction of energetically shallow traps for the charge carriers induced by the presence of the Rb ions. The shallow traps either allow for radiative recombination of the charge carriers or are sufficiently shallow that thermal phonons might induce detrapping, hence slowing down the recombination processes in the 2D-HOIP crystals. This effect is more prominent in BA_2_PbBr_4_, suggesting that Rb doping introduces more shallow traps, although not a higher amount of traps overall, in BA_2_PbBr_4_ than PEA_2_PbBr_4_. We note however that the decay time is still very fast at 10 K, with residual luminescence below 1% for all undoped and Rb-doped perovskites after 5 s at 10 K, and thus negligible at much higher temperatures, such as room temperature. This is further corroborated by the TL measurements shown in the inset of Figure 5a and Supplementary Figure S7, which show an increase in trap states inside the 2D-HOIP crystals. The fitting of the TL curves is presented in Supplementary Table S5. Undoped, PEA_2_PbBr_4_ features some traps at 10 meV but less pronounced than the Rb-doped variant. Undoped BA_2_PbBr_4_ shows no traps at all. Both BA_2_PbBr_4_ and PEA_2_PbBr_4_ show significant increases in the number of traps after doping. The trap energies are however very shallow, at 14 meV for Rb-doped BA_2_PbBr_4_ and between 10 and 40 meV for Rb-doped PEA_2_PbBr_4_. In addition, Rb-doped PEA_2_PbBr_4_ shows a higher overall amount of traps and deeper traps than Rb-doped BA_2_PbBr_4_, which might also explain the much stronger broadening of the PL spectrum of Rb-doped PEA_2_PbBr_4_. Comparing to the other Rb crystals, RbPb_2_Br_5_, the slower afterglow and more concentrated traps in Rb-doped BA_2_PbBr_4_ and PEA_2_PbBr_4_ are expected as RbPb_2_Br_5_ crystals have an average afterglow decay time of 427.6 s and a trap concentration of about 10^7^, see Supplementary Figure S8.

Supplementary Figure S9 shows the γ-ray scintillation decay curves of the undoped and Rb-doped 2D-HOIP crystals. The results of the exponential decay fitting are presented in Supplementary Table S6. Similarly to the observed PL decay curves, the presence of Rb leads to faster scintillation decay times at high γ-ray energies as well. Both Rb-doped BA_2_PbBr_4_ and PEA_2_PbBr_4_ show faster decay times than the undoped counterparts. The average decay times are 67.4 and 52.6 ns for undoped and Rb-doped BA_2_PbBr_4_, respectively, and 49.8 and 45.6 ns for undoped and Rb-doped PEA_2_PbBr_4_, respectively. The decay times are thus 15% and 8% faster for Rb-doped BA_2_PbBr_4_ and PEA_2_PbBr_4_, respectively. The effect of the Rb doping shortening the scintillation decay times is less than that of the TRPPL decay times due to the increase of the trap numbers with high-energy excitation, which is also observed by TL.^11^ The increase of the trap numbers would also lead to additional nonradiative processes, depopulating emissive species such as excitons, and thus faster time-resolved PL fast components. The shortening of the lifetime is also more prominent for Rb-doped BA_2_PbBr_4_, which also shows less PL broadening, and hence possibly more nonradiative traps. In contrast, although they are losses due to nonradiative processes, γ-ray PHS measurements show increases in the light yields of the Rb-doped 2D-HOIP crystals compared to the undoped ones. The PHS at 59.5 keV of the undoped and Rb-doped crystals are shown in Figure 5b. Additional PHS at 661.7 keV are shown in Supplementary Figure S10. Derived from the spectra, the improvements of the light yields at 59.5 keV due to Rb doping are 54% and 27% for BA_2_PbBr_4_ and PEA_2_PbBr_4_, respectively. The numbers are consistent with those observed at 661.7 keV of 62% and 22%, respectively (Supplementary Figure S10). On average, with an assumption of linear scintillation responses, the enhancements of the light yields for both BA_2_PbBr_4_ and PEA_2_PbBr_4_ are 58% and 25%, respectively. Since we observe the increase of the light yield following Rb doping, it could be that the presence of Rb ions also leads to the improvements from other characteristics in the 2D-HOIP scintillators, such as the improved charge carrier transport or quantum yield, which would rather improve the scintillation despite of the introduction of traps.^11^ This can be seen as the improvements of the light yields are not solely linear with the decrease of the band gaps as those in BA_2_PbBr_4_ and PEA_2_PbBr_4_ only decrease by 22% and 5%, respectively (see Figure 2). In comparison with other studies on 2D-HOIP crystals, this small ion doping technique still improves the light yield in comparison to those of one-half^54^ and complete divalent replacement.^55−58^

## Conclusion

We investigated the effect of Rb doping on the structural, optical, and scintillation properties of the 2D-HOIP BA_2_PbBr_4_ and PEA_2_PbBr_4_ crystals. Overall, we observe that Rb doping leads to lattice expansion of both crystals with a more prominent effect on BA_2_PbBr_4_. Due to this effect, both 2D-HOIP crystals showed a smaller band gap, which was confirmed by absorption measurements. However, the PEA_2_PbBr_4_ PL properties were more strongly affected, leading to a small shift in the peak emission and a significant broadening of the emission. This is reflected in the RL as well. Both crystals show broader RL spectra and the appearance of additional peaks, while this is more prominent in PEA_2_PbBr_4_. Both 2D-HOIP crystals also showed a faster time-resolved PL and scintillation decay time due to Rb doping and a slightly slower afterglow. The broadening and the change in the decay times can be explained by the introduction of traps via lattice distortion by the introduction of the relatively large Rb ions into the BA_2_PbBr_4_ and PEA_2_PbBr_4_ perovskite structure, which is corroborated by TL measurements. However, the introduction of Rb also leads to significantly higher light yields with increases of 58% and 25% for BA_2_PbBr_4_ and PEA_2_PbBr_4_, respectively. The improvements in light yield and scintillation decay times make Rb doping of the investigated 2D-HOIP crystals particularly interesting for fast timing imaging applications and radiation detection.

## Supporting Information Available

Reitveld refinements of single-crystal XRD, crystal parameters and properties of Rb-doped crystals and RbPb_2_Br_5_, absorption spectra from undoped and Rb-doped crystals with Elliot fits, comparison of RL and PL spectra for all crystals, glow curve fits, scintillation decay curves at 661.7 keV (^137^Cs), and pulse height spectra at 661.7 keV (PDF)
