# Supplementary material for: Lattice Expansion in Rb-Doped Hybrid Organic–Inorganic Perovskite Crystals Resulting in Smaller Band-Gap and Higher Light-Yield Scintillators
Source: Inorg Chem. 2023 May 26;62(23):8892–902. doi: 10.1021/acs.inorgchem.3c00270 (PMC10265701; doi:10.1021/acs.inorgchem.3c00270)
Supplement: Supplementary file 1 — ic3c00270_si_001.pdf [file ic3c00270_si_001.pdf]

Supplementary Information for Lattice Expansion in Rb Doped Hybrid Organic-Inorganic Perovskite Crystals Resulting Smaller-Bandgap and Higher-Light-Yield Scintillators

Francesco Maddalena,[*,†,‡] Muhammad Haris Mahyuddin,[¶] Dominik Kowal,[§] Marcin E. Witkowski,[‖] Michal Makowski,[‖] Md Abdul Kuddus Sheikh,[§] Somnath Mahato,[§] Roman Jędrzejewski,[§] Winicjusz Drozdowski,[‖] Christophe Dujardin,[⊥] Cuong Dang,[†,‡] and Muhammad Danang Birowosuto[*,§]

†*School of Electrical and Electronic Engineering, Nanyang Technological University, Singapore 639798, Singapore*

‡*CINTRA UMI CNRS/NTU/THALES 3288, Research Techno Plaza, 50 Nanyang Drive, Border X Block, Level 6, Singapore 637553, Singapore*

¶*Research Group of Advanced Functional Materials, Faculty of Industrial Technology, Institut Teknologi Bandung, Bandung 40132, Indonesia.*

§*Łukasiewicz Research Network-PORT Polish Center for Technology Development, Stabłowicka 147, 54-066 Wrocław, Poland*

‖*Institute of Physics, Faculty of Physics, Astronomy, and Informatics, Nicolaus Copernicus University in Torun, ul. Grudziadzka 5, 87-100 Torun, Poland*

⊥*Universitè de Lyon, Universitè Claude Bernard, Lyon 1, CNRS, Institut Lumière Matière UMR5306, Villeurbanne F-69622, France.*

E-mail: francesco_maddalena@ntu.edu.sg; muhammad.birowosuto@port.lukasiewicz.gov.pl

List of Figures

1 Rietveld refinements of single-crystal X-ray diffraction (XRD) spectra (right) and crystal structure (left) from a) undoped BA_2PbBr_4, b) Rb-doped BA_2PbBr_4, c) undoped PEA_2PbBr_4, and d) Rb-doped PEA_2PbBr_4 using reference structures from.[1] The lattice parameters are shown in Supplementary Table S1. 5

2 $RbPb_2Br_5$ crystals as comparisons. a) Images of crystals, b) Reitveld refinements of XRD spectrum using reference from,[2] The lattice parameters are shown in Supplementary Table S1. c) Photoluminescence (PL) and absorption spectra, d) Temperature-dependent Radioluminescence (RL) and light yield as function of temperature (inset). 6

3 X-ray photo-electron spectroscopy data for the Rb-doped 2D-perovskites: Pb-peaks for a) Rb-BA_2PbBr_4 and b) Rb-PEA_2PbBr_4; C-peaks for c) Rb-BA_2PbBr_4 and d) Rb-PEA_2PbBr_4; N-peaks for e) Rb-BA_2PbBr_4 and f) Rb-PEA_2PbBr_4, and Br-peaks for g) Rb-BA_2PbBr_4 and h) Rb-PEA_2PbBr_4. 8

4 Raman spectra and their vibrational modes similar as reported in[3] for undoped, Li doped, and Rb doped, a) BA_2PbBr_4 and b) PEA_2PbBr_4. The weak influence of doping to the Raman spectra for PEA_2PbBr_4 can be related with the structure itself or unexpected small concentration. 9

5 Absorption spectra from a) undoped and b) Rb-doped BA_2PbBr_4 and c) undoped and d) Rb-doped PEA_2PbBr_4 and their fitting curves with Elliot method in Supplementary Eqs. S1 and S2.[4] 10

6 PL (solid lines) and RL (dotted lines) spectra for a) undoped (black) and Rb doped (red) BA_2PbBr_4 and c) undoped (black) and d) Rb-doped (red) PEA_2PbBr_4. 11

7 The fit of glow curves of undoped and Rb-doped BA_2PbBr_4 and PEA_2PbBr_4 with multiple Randal-Wilkins method in Supplementary Eq. SS4.[5,6] The parameters of the fit are shown in Supplementary Table S5. 12

8 $RbPb_2Br_5$ crystals as comparisons. a) Afterglow curve and b) TL peaks with the parameters are on the inset table. 13
9 γ-ray excited scintillation decay curves at 661.7 keV (^{137}Cs) and room temperature for a) undoped and b) Rb-doped BA_2PbBr_4 and c) undoped and d) Rb-doped PEA_2PbBr_4 and their fitting curves with three exponential decay model. 14
10 Pulse height spectra with a 661.7 keV gamma-ray sources for undoped and Rb-doped BA_2PbBr_4 and PEA_2PbBr_4. The arrows indicate the position of the photopeaks and the positions of the undoped peaks were normalized to each other for showing the differences with the doped ones. 15

List of Tables

1 Crystal data and structure refinement from XRD spectra of undoped and Rb-doped BA_2PbBr_4 and PEA_2PbBr_4 measured at ambient pressure. 7
2 Parameters of the PL decay curves, where τ_i is the decay time, C_i is the contribution of the decay time and $\bar{\tau}$ is the mean time of the decay. 9
3 Parameters for the negative thermal quenching fitting. 10
4 Parameters of the afterglow curves, where τ_i is the decay time, C_i is the contribution of the decay time and $\bar{\tau}$ is the mean time of the decay. 11
5 Parameters of the thermoluminescence (TL) peak fitting, where T_{max} is temperature where the maximum of the peak occurs, E is the trap depth, n_0 is the trap concentration and σ is the frequency factor. 13
6 Parameters of the scintillation decay curves, where τ_i is the decay time, C_i is the contribution of the decay time and $\bar{\tau}$ is the mean time of the decay. 14

Rietveld Refinements

The Rietveld program Fullprof[7] was selected to analyse the data in this study. The profile function of a Thompson-Cox-Hastings pseudo-Voigt function was used. The background function was the sixth order of polynomials. The results are shown in Supplementary Figures 1 and 2 while the parameters are shown in Supplementary Table 1.

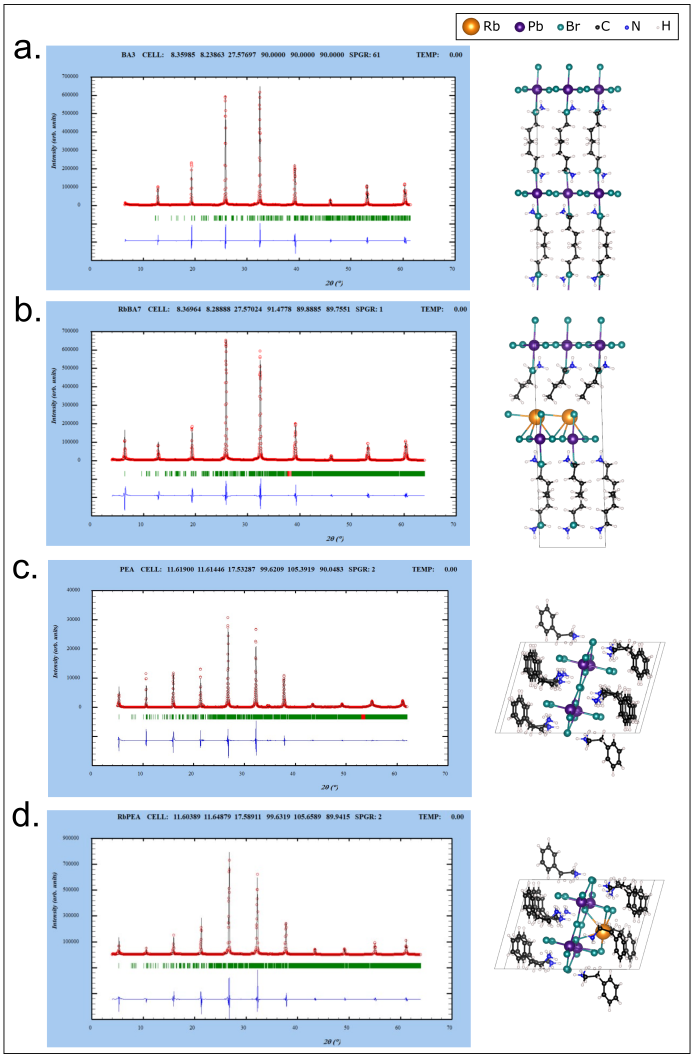

Supplementary Figure S 1: Rietveld refinements of single-crystal X-ray diffraction (XRD) spectra (right) and crystal structure (left) from a) undoped BA_2PbBr_4, b) Rb-doped BA_2PbBr_4, c) undoped PEA_2PbBr_4, and d) Rb-doped PEA_2PbBr_4 using reference structures from.[1] The lattice parameters are shown in Supplementary Table S1.

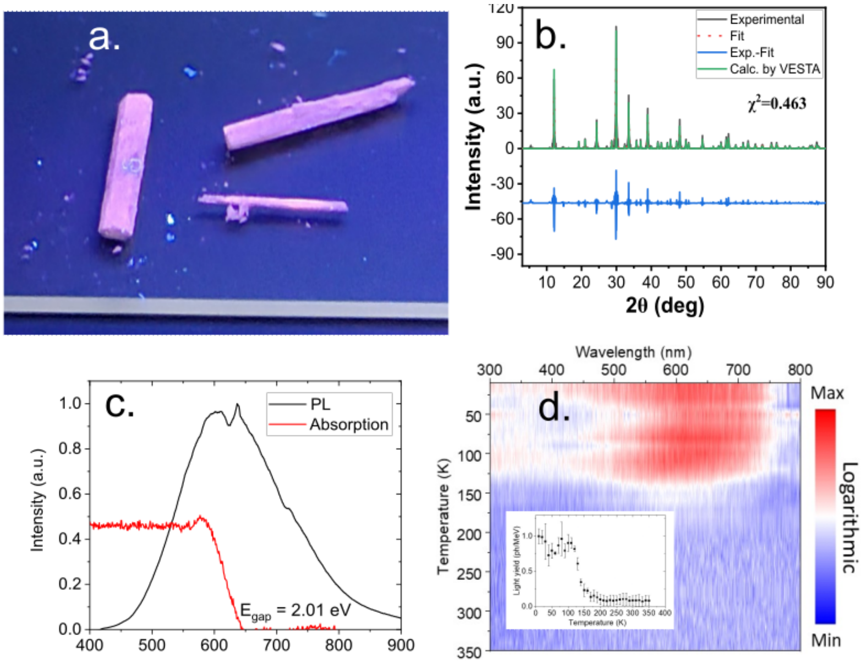

Supplementary Figure S 2: $RbPb_2Br_5$ crystals as comparisons. a) Images of crystals, b) Reitveld refinements of XRD spectrum using reference from,[2] The lattice parameters are shown in Supplementary Table S1. c) Photoluminescence (PL) and absorption spectra, d) Temperature-dependent Radioluminescence (RL) and light yield as function of temperature (inset).

Supplementary Table S 1: Crystal data and structure refinement from XRD spectra of undoped and Rb-doped BA_2PbBr_4 and PEA_2PbBr_4 measured at ambient pressure.

**Lattice parameters**	BA_2PbBr_4	Rb-BA_2PbBr_4	PEA_2PbBr_4	Rb-PEA_2PbBr_4	$RbPb_2Br_5$
Source			Laboratory X-ray (Cu Kα)		
Wavelength			1.540(6) Å		
Chemical Formula	$C_8H_{24}Br_4N_2Pb$	$C_{24}H_{72}Br_{16}N_6Pb_4Rb_2$	$C_{16}H_{24}Br_4N_2Pb$	$C_{56}H_{84}Br_{16}N_7Pb_4Rb$	$RbPb_2Br_5$
Formula weight	675.12 g/mol	679.39 g/mol	771.20 g/mol	775.47 g/mol	899.39 g/mol
Temperature	298(2) K	298(2) K	298(2) K	298(2) K	298(2) K
Crystal system	Orthorhombic	Triclinic	Triclinic	Triclinic	Body-centered cubic
Space group (No.)	$Pbca$ (61)	$P1$ (1)	$P-1$ (2)	$P-1$ (2)	$I4/mcm$ (140)
Unit cell	a = 8.359(9) Å	a = 8.369(6) Å	a = 11.619(0) Å	a = 11.603(9) Å	a = 8.450(2) Å
dimensions	b = 8.238(6) Å	b = 8.288(9) Å	b = 11.614(5) Å	b = 11.648(8) Å	b = 8.450(2) Å
	c = 27.577(0) Å	c = 27.570(2) Å	c = 17.532(9) Å	c = 17.589(1) Å	c = 14.597(7) Å
	α = 90.00(0) °	α = 91.47(8) °	α = 99.62(1) °	α = 99.63(2) °	α = 90.00(0) °
	β = 90.00(0) °	β = 89.88(8) °	β = 105.39(2) °	β = 105.65(9) °	β = 90.00(0) °
	γ = 90.00(0) °	γ = 89.75(5) °	γ = 90.04(8) °	γ = 89.94(2) °	γ = 90.00(0) °
Volume	1899.328(3) $Å^3$	1912.026(3) $Å^3$	2246.541(6) $Å^3$	2254.588(3) $Å^3$	1042.351(3) $Å^3$
Z	4	4	4	4	4
d_{hkl}	d_{002} = 13.788(5) Å	d_{002} = 13.783(0) Å	d_{001} = 16.648(1) Å	d_{001} = 16.681(9) Å	d_{002} = 7.298(5) Å
χ^2	2.9	4.8	3.7	4.2	1.3
R_p	16.2	18.5	24.3	26.9	34.9
R_{wp}	23.0	27.2	35.0	38.4	42.3
CCDC No.	2257540	2257555	2257548	2238869	2239005
Calculated density	2.361 g/cm^3	2.364 g/cm^3	2.280 g/cm^3	2.285 g/cm^3	5.734 g/cm^3
Rb/Pb precursor	Undoped	5:100	Undoped	5:100	Undoped
XPS Rb/Pb (%)	-	2.7%	-	4.6%	-
ICPMS Rb/Pb* (%)	0.5/14,000 ($\ll$0.1%)	435:13,700 (3.2%)	0.1/13,800 ($\ll$0.1%)	121:3,150 (3.8%)	-

*Inductively coupled plasma mass spectrometry Rb/Pb ratio with concentrations in parts per billion (ppb)

Absorption Curve fitting

The fit was performed by using Elliot formalism.[8] In principle, the contributions to the absorption coefficient (α) can be defined from free carriers (continuum) (α_c) and excitons (α_{ex}).

$$\alpha(\hbar\omega) = \alpha_c + \alpha_{ex} \tag{S1}$$

$$\alpha(\hbar\omega) = P_{cv}\left[\theta(\hbar\omega - E_g)\cdot\left(\frac{\pi e^{\pi x}}{sinh(\pi x)}\right) + R_{ex}\sum_{n=1}^{\infty}\frac{4\pi}{n^3}\cdot\delta(\hbar\omega - E_g + \frac{R_{ex}}{n^2})\right] \tag{S2}$$

Where the frequency dependence of P_{cv} is approximated as a constant and related to the interband transition matrix element, $\hbar\omega$ is the photon energy, $\theta(\hbar\omega$ - $E_g)$ is the Heaviside step function, x is defined as $\sqrt{R_{ex}/(\hbar\omega - E_g)}$, and δ denotes a delta function. R_{ex} is exciton Rydberg energy; n is the principle quantum number. The fits to the absorption curves are shown in Supplementary Fig. S5.

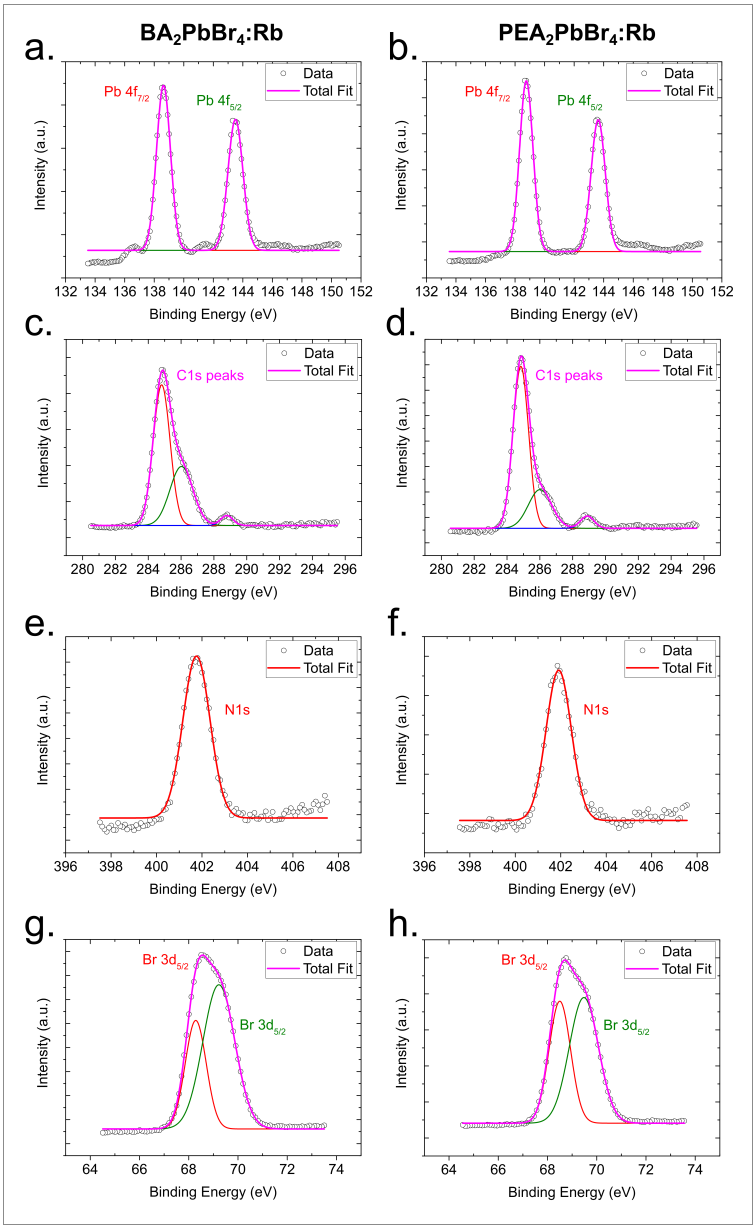

Supplementary Figure S 3: X-ray photo-electron spectroscopy data for the Rb-doped 2D-perovskites: Pb-peaks for a) Rb-BA_2PbBr_4 and b) Rb-PEA_2PbBr_4; C-peaks for c) Rb-BA_2PbBr_4 and d) Rb-PEA_2PbBr_4; N-peaks for e) Rb-BA_2PbBr_4 and f) Rb-PEA_2PbBr_4, and Br-peaks for g) Rb-BA_2PbBr_4 and h) Rb-PEA_2PbBr_4.

Time-resolved PL fitting

PL decay curves were fitted with three exponential decay model and the parameters are shown in Supplementary Table 2.

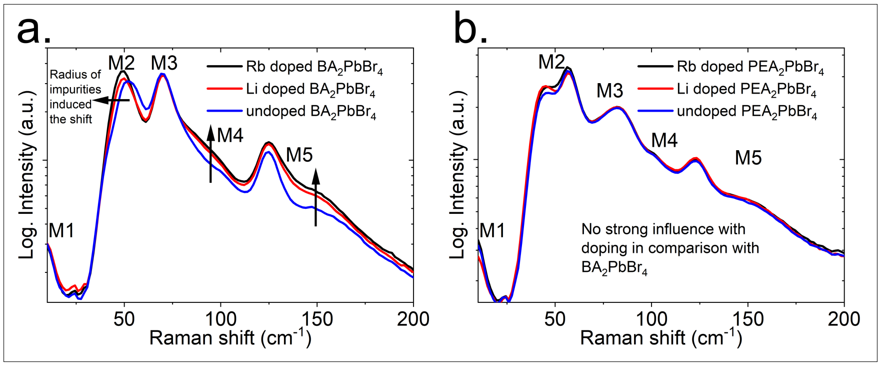

Supplementary Figure S 4: Raman spectra and their vibrational modes similar as reported in[3] for undoped, Li doped, and Rb doped, a) BA_2PbBr_4 and b) PEA_2PbBr_4. The weak influence of doping to the Raman spectra for PEA_2PbBr_4 can be related with the structure itself or unexpected small concentration.

Supplementary Table S 2: Parameters of the PL decay curves, where τ_i is the decay time, C_i is the contribution of the decay time and $\bar{\tau}$ is the mean time of the decay.

Compound	τ_1 (ns)	C_1 (%)	τ_2 (ns)	C_2 (%)	τ_3 (ns)	C_3 (%)	$\bar{\tau}$ (ns)
BA_2PbBr_4	5.2 ± 0.1	46	34.6 ± 0.3	33	289.7 ± 8.6	21	74.6 ± 1.1
Rb-BA_2PbBr_4	4.7 ± 0.1	49	25.4 ± 0.9	35	250.2 ± 3.9	16	51.2 ± 0.9
PEA_2PbBr_4	7.8 ± 0.1	68	32.1 ± 0.2	24	539.7 ± 15.7	8	56.2 ± 1.4
Rb-PEA_2PbBr_4	7.4 ± 0.1	69	28.9 ± 0.2	24	423.6 ± 12.3	7	41.7 ± 1.5

The shift of PL and RL spectra after Rb doping

PL and RL spectra of undoped and Rb-doped BA_2PbBr_4 and PEA_2PbBr_4crystals are shown in Supplementary Fig. 6. The shift to red is more significant for Rb-doped PEA_2PbBr_4 crystals. This is because the strong self absorption due to the small Stokes shift of Rb-doped PEA_2PbBr_4 of 0.11 eV. For Rb-doped BA_2PbBr_4, the Stokes shift is still about 0.20 eV.

Temperature-dependent RL fitting

The fit was carried out according to the model proposed by Shibata et al.:[9]

$$\frac{I(T)}{I(0)} = \frac{1 + D \cdot e^{-E/k_B T}}{1 + C \cdot e^{-E_1/k_B T}} \quad \text{(S3)}$$

where D is the negative thermal quenching coefficient which describes the contribution from thermally excited electrons, C is the thermal quenching coefficients related to non-radiative

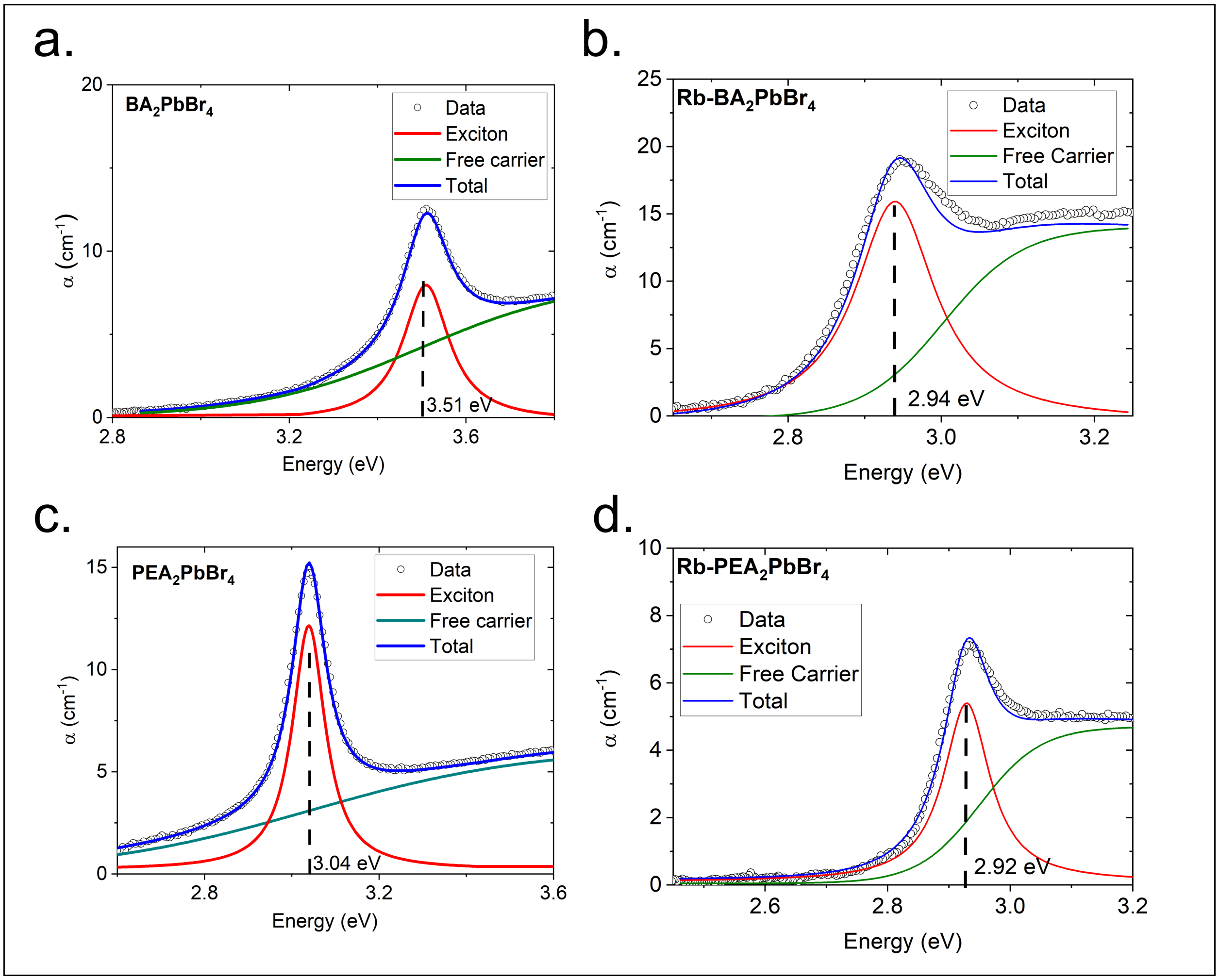

Supplementary Figure S 5: Absorption spectra from a) undoped and b) Rb-doped BA_2PbBr_4 and c) undoped and d) Rb-doped PEA_2PbBr_4 and their fitting curves with Elliot method in Supplementary Eqs. S1 and S2.[4]

electrons excitation leading to thermal quenching, E is the activation energy for negative thermal quenching and E_1 is the activation energies for typical thermal quenching, respectively and k_B is the Boltzmann constant. The parameters are shown in Supplementary Table 3.

Supplementary Table S 3: Parameters for the negative thermal quenching fitting.

Compound	C (-)	E_1 (meV)	D (-)	E (meV))
BA_2PbBr_4	5.98×10^4	277	1.91×10^3	144
Rb-BA_2PbBr_4	7.91×10^5	322	5.56×10^4	209
PEA_2PbBr_4	1.31×10^7	438	3.27×10^5	304
Rb-PEA_2PbBr_4	6.19×10^5	411	342	141

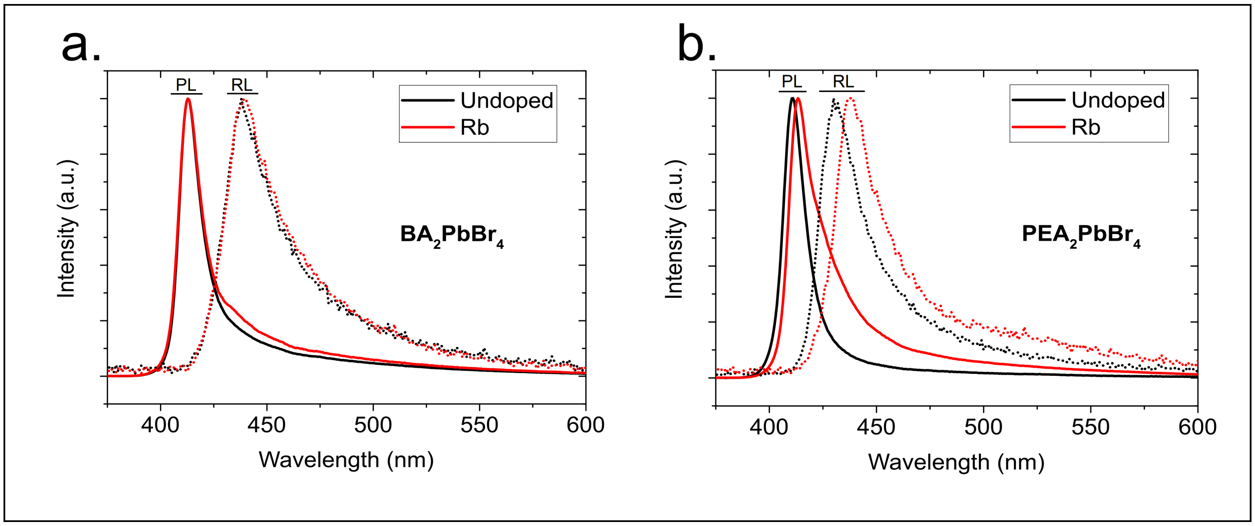

Supplementary Figure S 6: PL (solid lines) and RL (dotted lines) spectra for a) undoped (black) and Rb doped (red) BA_2PbBr_4 and c) undoped (black) and d) Rb-doped (red) PEA_2PbBr_4.

Afterglow fitting

The afterglow curves in Figure 4a were fitted with two exponential decay model. The parameters are shown in Supplementary Table 4

Supplementary Table S 4: Parameters of the afterglow curves, where τ_i is the decay time, C_i is the contribution of the decay time and $\bar{\tau}$ is the mean time of the decay.

Compound	τ_1 (s)	C_1 (%)	τ_2 (s)	C_2 (%)	$\bar{\tau}$ (s)
BA_2PbBr_4	5.6	100	0	0	5.6
Rb-BA_2PbBr_4	10.3	83	82.2	17	22.3
PEA_2PbBr_4	0.9	100	0	0	0.9
Rb-PEA_2PbBr_4	2.4	95	52.7	5	5.1

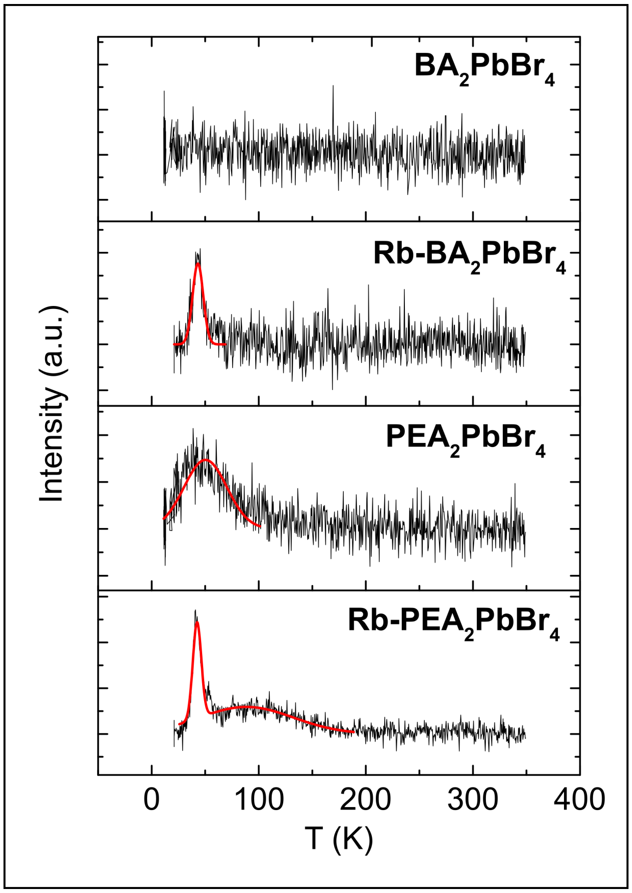

Supplementary Figure S 7: The fit of glow curves of undoped and Rb-doped BA_2PbBr_4 and PEA_2PbBr_4 with multiple Randal-Wilkins method in Supplementary Eq. SS4.[5,6] The parameters of the fit are shown in Supplementary Table S5.

Glow curve fitting

For the quantitative analysis, we deconvolute the glow curves into k glow peaks, based on the classic Randall-Wilkins equation:[5,6]

$$I_{TL} = \sum_{i=1}^{k} n_{0_i} V \sigma_i \exp\left(-\frac{E_i}{k_B T}\right) \exp\left(-\frac{\sigma_i}{\beta}\int_{T_0}^{T} \exp\left(-\frac{E_i}{k_B T'}\right) dT'\right) \tag{S4}$$

where T is the temperature, β is the heating rate, k_B is the Boltzmann constant, n_{0_i} is the initial trap concentration, V is the crystal volume, E_i is the trap depth, and σ_i is the frequency factor of each component. The unit-less $n_{0_i}V$ or A_i is used to compare afterglow of different crystals. From the fits of Supplementary Eq. S4 to Supplementary Fig. 7, we obtain parameters

as shown in Supplementary Table 5.

Supplementary Table S 5: Parameters of the thermoluminescence (TL) peak fitting, where T_{max} is temperature where the maximum of the peak occurs, E is the trap depth, n_0 is the trap concentration and σ is the frequency factor.

Compound	T_{max} (K)	E (meV)	n_0 (a.u.)	σ (s^{-1})
BA_2PbBr_4	No trap			
Rb-BA_2PbBr_4	43	14	7.2×10^3	3.9×10^3
PEA_2PbBr_4	50	10	6.0×10^3	3.9×10^3
Rb-PEA_2PbBr_4	42	13	5.0×10^4	2.0×10^4
	88	39	5.1×10^3	5.7×10^3

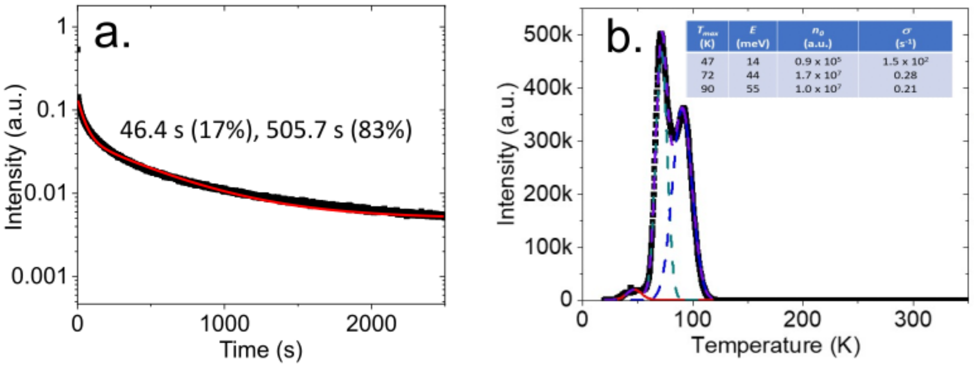

Supplementary Figure S 8: $RbPb_2Br_5$ crystals as comparisons. a) Afterglow curve and b) TL peaks with the parameters are on the inset table.

Scintillation decay fitting

Scintillation decay curves in Supplementary Fig. 9 were fitted with three exponential decay model and the parameters are shown in Supplementary Table 6

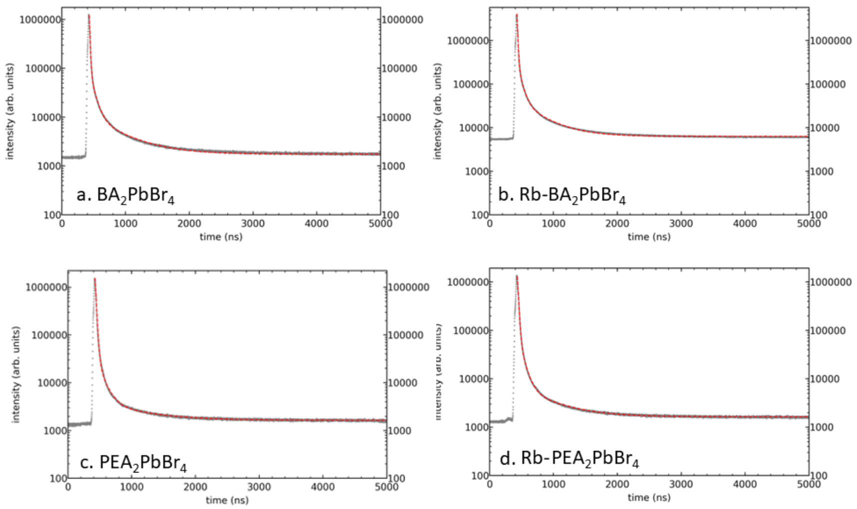

Supplementary Figure S 9: γ-ray excited scintillation decay curves at 661.7 keV (^{137}Cs) and room temperature for a) undoped and b) Rb-doped BA_2PbBr_4 and c) undoped and d) Rb-doped PEA_2PbBr_4 and their fitting curves with three exponential decay model.

Supplementary Table S 6: Parameters of the scintillation decay curves, where τ_i is the decay time, C_i is the contribution of the decay time and $\bar{\tau}$ is the mean time of the decay.

Compound	τ_1 (ns)	C_1 (%)	τ_2 (ns)	C_2 (%)	τ_3 (ns)	C_3 (%)	$\bar{\tau}$ (ns)
BA_2PbBr_4	4.7 ± 0.1	72.6	70.7 ± 0.2	16.4	476.1 ± 4.0	11.0	67.4 ± 1.2
Rb-BA_2PbBr_4	4.4 ± 0.1	73.6	59.6 ± 0.2	15.6	371.4 ± 3.3	10.8	52.6 ± 1.1
PEA_2PbBr_4	13.4 ± 0.1	85.4	98.2 ± 1.3	10.1	632.9 ± 10.2	4.5	49.8 ± 8.9
Rb-PEA_2PbBr_4	12.3 ± 0.1	81.5	74.6 ± 1.4	13.3	493.4 ± 24.7	5.2	45.6 ± 4.5

Pulse height spectra

Here we attached additional pulse height spectra (PHS) measured with 661.7 keV gamma-ray sources as shown in Supplementary Fig. 10. Derived from those spectra, the improvements of the light yields due to the Rb doping are 1.62 and 1.22 folds for BA_2PbBr_4 and PEA_2PbBr_4, respectively.

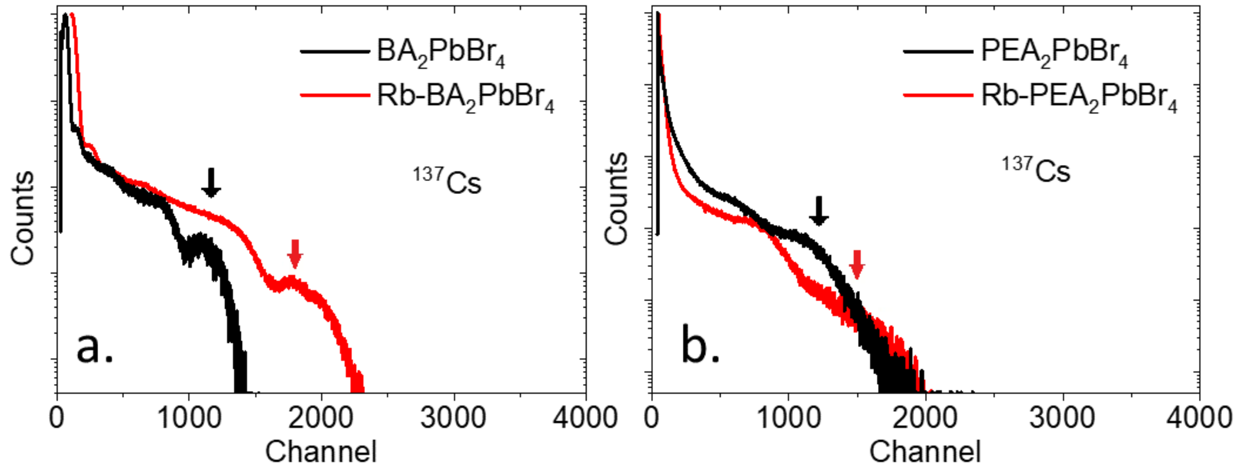

Supplementary Figure S 10: Pulse height spectra with a 661.7 keV gamma-ray sources for undoped and Rb-doped BA_2PbBr_4 and PEA_2PbBr_4. The arrows indicate the position of the photopeaks and the positions of the undoped peaks were normalized to each other for showing the differences with the doped ones.

References

(1) Maddalena, F.; Xie, A.; Arramel,; Witkowski, M. E.; Makowski, M.; Mahler, B.; Drozdowski, W.; Mariyappan, T.; Springham, S. V.; Coquet, P.; Dujardin, C.; Birowosuto, M. D.; Dang, C. *J. Mater. Chem. C* **2021**, *9*, 2504–2512.

(2) Abia, C.; López, C. A.; Gainza, J.; Rodrigues, J. E. F. S.; Ferrer, M. M.; Nemes, N. M.; Dura, O. J.; Martínez, J. L.; Fernández-Díaz, M. T.; Álvarez Galván, C.; Németh, G.; Kamarás, K.; Fauth, F.; Alonso, J. A. *J. Mater. Chem. C* **2022**, *10*, 6857–6865.

(3) Dhanabalan, B.; Leng, Y.-C.; Biffi, G.; Lin, M.-L.; Tan, P.-H.; Infante, I.; Manna, L.; Arciniegas, M. P.; Krahne, R. *ACS Nano* **2020**, *14*, 4689–4697.

(4) Diguna, L. J.; Jonathan, L.; Mahyuddin, M. H.; Arramel,; Maddalena, F.; Mulyani, I.; Onggo, D.; Bachiri, A.; Witkowski, M. E.; Makowski, M.; Kowal, D.; Drozdowski, W.; Birowosuto, M. D. *Mater. Adv.* **2022**, *3*, 5087–5095.

(5) Birowosuto, M. D.; Cortecchia, D.; Drozdowski, W.; Brylew, K.; Lachmanski, W.; Bruno, A.; Soci, C. *Sci. Rep.* **2016**, *6*, 37254.

(6) Randall, J. T.; Wilkins, M. H. F. *Proc. R. Soc. Lond. A* **1945**, *184*, 365–389.

(7) Rodríguez-Carvajal, J. *Phys. B: Condens. Matter* **1993**, *192*, 55–69.

(8) Elliott, R. J. *Phys. Rev.* **1957**, *108*, 1384–1389.

(9) Shibata, H. *Jpn. J. Appl. Phys.* **1998**, *37*, 550–553.
